# Air pollution in Bishkek, Kyrgyzstan: Driving factors and state response

**DOI:** 10.1002/puh2.22

**Published:** 2022-10-17

**Authors:** Kenesh O. Dzushupov, Julian M. A. Buban, Arsen A. Aidaraliev, Attaullah Ahmadi, Priyanka Chahal, Muiz Ibrahim, Xu Lin, M. B. N. Kouwenhoven

**Affiliations:** ^1^ Department of Public Health International School of Medicine Bishkek Kyrgyzstan; ^2^ College of Medicine University of the Philippines Manila Philippines; ^3^ Office of the University President International University of Kyrgyzstan Bishkek Kyrgyzstan; ^4^ Department of Public Health Kazakh National Medical University Almaty Kazakhstan; ^5^ S. Tentishev Asian Medical Institute Kant Kyrgyzstan; ^6^ International School of Medicine International University of Kyrgyzstan Bishkek Kyrgyzstan; ^7^ Department of Thoracic Surgery The First Affiliated Hospital School of Medicine Zhejiang University Hangzhou Zhejiang China; ^8^ Department of Physics Xi'an Jiaotong‐Liverpool University Suzhou China

**Keywords:** air pollution, Bishkek, disease burden, health, Kyrgyzstan

## Abstract

The present article aims to describe the status quo of the atmospheric air quality in Bishkek and the state measures taken to improve it and to give the perspective of research and policy development. Air pollution is one of the major environmental risks for premature death from respiratory diseases, cancer, strokes, heart attacks, diabetes, and other diseases. It exerts a negative effect on worker productivity and mental health. In the last 30 years, Bishkek, the capital of the Kyrgyz Republic, has turned from one of the cleanest and greenest cities in the former Soviet Union to one of the most polluted cities in the world. The roots of that transformation lie in the negative socio‐economic changes taking place in the country, including the population doubling of Bishkek mainly due to internal migration, uncontrolled construction of houses without relevant infrastructure, worsening socio‐economic conditions, increased number of used vehicles, and low quality of gasoline. The main sources of air pollution in Bishkek are domestic heating and vehicle exhaust fumes. During the winter, air pollution is aggravated by frequent temperature inversion and air stagnation due to air trapping by high‐rise buildings. The state's approaches and measures to address this issue are reflected in its laws and policies. The city and national government have taken a range of strategic measures to transform Bishkek into a green city with a favourable environment. Recommendations on research and policy development are provided in this perspective.

## INTRODUCTION

Humanity has been aware of the essential role of air in human health since ancient times. Even during those times, the Greek and Roman contemporaries of Hippocrates complained of the troublesome effects of burning coal and bemoaned sooty and stinky air [1], Nowadays, air pollution is among the top five leading risk factors for death globally, contributing to nearly 6.67 million deaths worldwide in 2019 [2]. It is one of the most significant environmental risks for premature death from respiratory diseases, cancer, strokes, heart attacks, diabetes, neurological conditions, and other diseases. It negatively affects worker productivity and mental health and has adverse birth outcomes.

**PHOTO 1:**
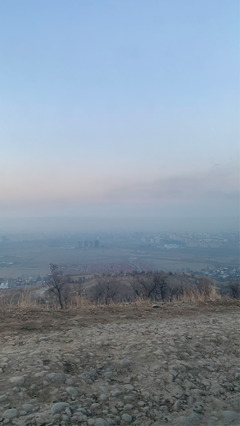
Smoggy Bishkek in early spring 2021 (Photo of Atai Oskonbaev, used with permission)

Studies have shown that air quality has improved in many high‐income countries, while dangerous levels of air pollution persist in low‐ and middle‐income countries [2]. Bishkek, the capital of Kyrgyzstan, a lower middle‐income country, has frequently appeared in the top 10 most polluted cities in the World Air Quality ranking. Recently developed air quality monitoring platforms often present Bishkek as having one of the world's highest levels of air pollution, particularly in the winter. During winter, the services collecting data from sensors installed in the city, often show dangerous levels of pollutants. Inhabitants are often recommended to avoid sports outside, to wear masks, to keep windows closed, and to use air purifiers indoors. During Soviet times, the capital of the Kyrgyz Republic was considered one of the greenest, cleanest, and most picturesque cities in the Soviet Union, with sanatoriums and resorts within its suburbs. Nowadays, a black ring of smoke often obscures the panoramic view of Bishkek from the snow‐white slopes. The present article aims to describe the status quo of the ambient air quality in Bishkek, and the state measures are taken to improve it and to give the perspective of research and policy development (Photo 1).

## PERSPECTIVE

### Air pollution and health

Air pollution is the contamination of ambient air by any chemical and physical substance of natural or man‐made origin [3]. Pollutants such as particulate matter, ozone, carbon monoxide and dioxide, nitrogen oxide, sulphur dioxide, volatile organic compounds (VOCs), dioxins, heavy metals and polycyclic aromatic hydrocarbons (PAHs) are considered harmful to humans. The most common air pollutant is fine particulate matter, defined as having a diameter of 2.5 microns or less (PM_2.5_). These particles can accumulate in the lungs and subsequently spread through the blood to other organs. Nearly all such inhaled airborne pollutants directly enter the bloodstream, bypassing detoxification in the liver and exerting a more direct and substantial effect on tissues and organs than toxins that enter through the stomach. Therefore, human health largely depends on air quality.

The effect of particulate pollution on life expectancy is greater than that of devastating infectious diseases such as tuberculosis and HIV/AIDS, behavioural killers like cigarette smoking, and even war [4]. Air pollution is one of the main risk factors for chronic obstructive pulmonary disease [5], pneumonia [6 ^,^ 7], paediatric respiratory infections [8 ^‐^ 10], acute respiratory distress syndrome [11 ^,^ 12], lung cancers [13 ^,^ 14], heart diseases [15 ^‐^ 16], stroke [17 ^‐^ 21], endocrine diseases [22 ^‐^ 24], and neurologic disorders [25 ^‐^ 27]. Air pollution is a risk factor linked to decreased cognitive function, diminished intelligence quotient, shortened attention span, and attention deficit hyperactivity disorder [28].

**FIGURE 1:**
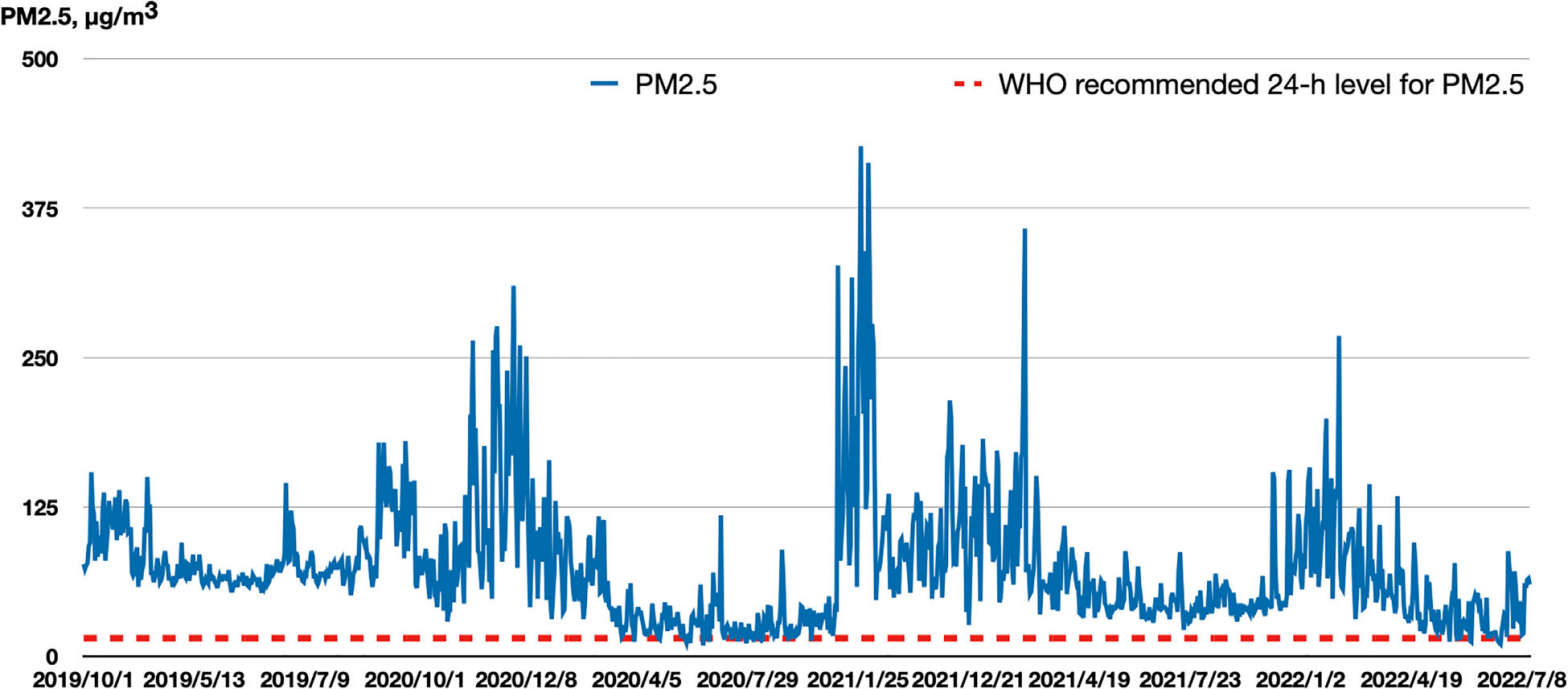
Air quality in Bishkek based on PM2.5 (microgram/m^3^) data from the Bishkek US Embassy station, from October 1, 2019 to August 5, 2022. (*Source*: Air Quality Historical Data Platform. https://aqicn.org/map/bishkek/ru/)

**FIGURE 2:**
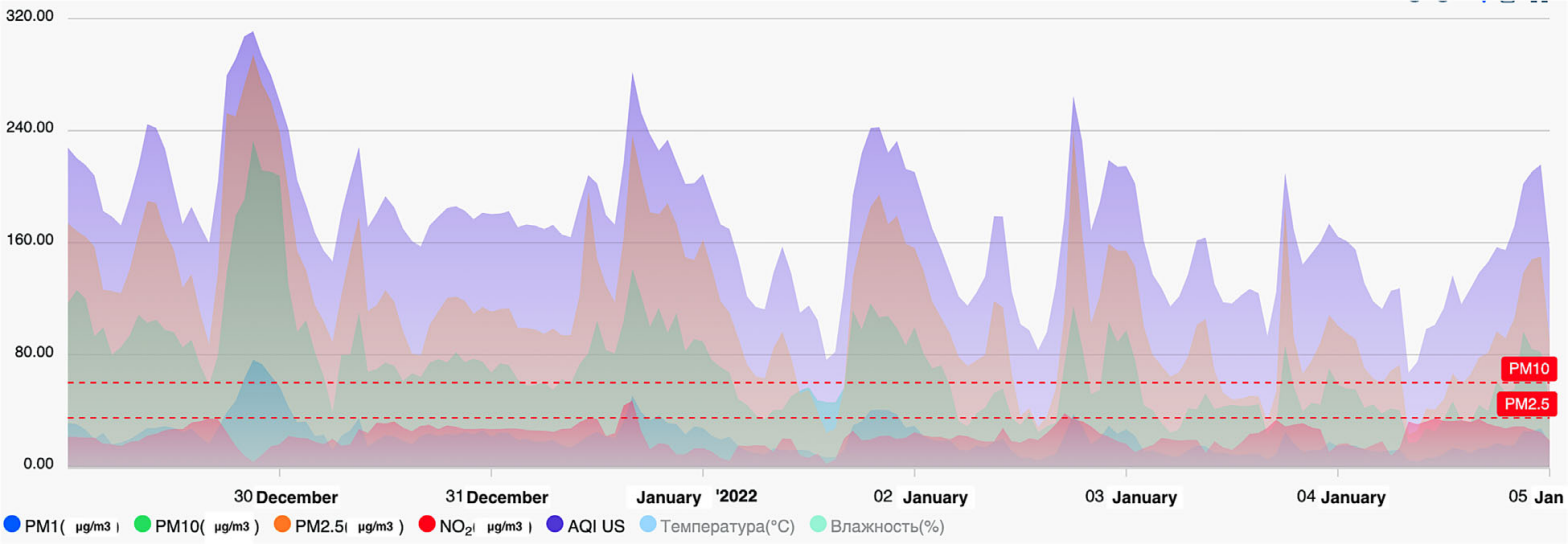
Air quality in Bishkek for a week of 29/12/2021‐05/01/2022 (Data of NGO MoveGreen, http://data.movegreen.kg/air)

In 2021, the WHO halved the highest recommended average emission level for PM2.5 from 10 to merely 5 μg/m^3^. The 24‐h level changed from 25 μg/m^3^ in 2005 to 15 μg/m^3.^ [29] Figure 1 shows the 24‐h PM2.5 average levels for 43 months in Bishkek, from October 2019 to July 2022. These concentrations are much higher than the recommended WHO level during most of the year. The seasonal component in the PM2.5 level is significant: in summer, the average level of pollution decreases, while it rises dramatically in winter. Table 1 presents the average 24‐h concentrations of other primary ambient air pollutants for December 2020‐February 2021. All air pollutants exceeded the WHO AQG levels and Kyrgyzstani maximum permissible levels at all analysed monitoring devices. The most polluted month in winter was December. Figure 2 presents average 24‐h levels of PM1, PM2.5, PM10, NO2, and AQI from 46 stations in Bishkek for the period of December 29, 2021, to January 5, 2022.

**TABLE 1:** Average 24‐h levels of several ambient air pollutants in Bishkek

	WHO AQG level, μg/m^3^	December 2020	January 2021	February 2021
SO2 (μg/m^3^)	20	57	73	31
NO2 (μg/m^3^)	25	99	140	84
NO (μg/m^3^)	50^a^	255	310	356
Formaldehyde (μg/m^3^)	3^a^	8	12	15
PM10 (μg/m^3^)	45	331	418	275

^a^Maximum permissible levels established in Kyrgyzstan.

The data shows that Bishkek's air quality is hazardous to the health of its residents. Between 2015 and 2019, the respiratory diseases incidence among Bishkek residents increased by 30.6% (from 19331.7 to 25238.1 per 100,000). In this period, the incidence of newly registered bronchial asthma almost doubled from 24 to 47 per 100,000, and lung cancer increased from 53 to 74 per 100,000. At that time, the officials reported a threefold increase in respiratory diseases among children under the age of 14, compared to that of adults or adolescents [30]. Ten and Sharshenova found a strong relationship between ambient air SO_2_ levels and incidences of respiratory diseases (*r* = 0.86) and neoplasms (*r* = 0.77) among children in Bishkek. They showed a direct positive correlation between the formaldehyde concentrations in the atmospheric air and the incidence of endocrine system diseases in children (*r* = 1.0) [31]. A study of 403 pregnant women, permanent residents of Bishkek, showed a sixfold increase in relative risk, and tenfold increase of additional risk in the development of somatic pathology and more than twice increased risk of the development of gestational complications in women living in the city areas with significant air pollution [32].

The ambient air pollution burden for Kyrgyzstan is estimated to have been responsible 49 attributable deaths and 1275 disability‐adjusted life‐years (DALYs) per 100,000 population for 2018 [33]. In Kyrgyzstan, the contribution of air pollution to the value of DALYs was estimated at 8%–10% [34]. Air pollution causes more than 2500 premature deaths, and is annually estimated to cause approximately 1.4 billion US$ in health‐related damage in Kyrgyzstan [35].

Air pollution‐related illnesses and premature deaths result in lost productivity and economic costs. In 2015, the estimated economic cost of premature deaths from air pollution was 3.571 billion Kyrgyzstani som, about 24% of Kyrgyzstan's GDP (in PPP$) [36]. The cost of lost productivity caused by diseases due to air pollution was estimated at 31 million US$, or 0.5% of the GDP, while the total economic burden was 388 million US$, or about 6% of the GNI [37]. The economic costs of air pollution (excluding costs related to diseases and consequences of indoor air pollution) in Bishkek are estimated between 0.4% and 2.6% of the GDP [38].

### Causes and sources of air pollution in Bishkek

During winters, the primary sources of air pollution in Bishkek are domestic heating and coal‐based power stations (CPSs). Residential heating devices are generally more harmful than CPSs because they employ low‐temperature fuel combustion, where a significant part of the fuel is not converted into heat and harmless gases, but harmful and toxic gases such as carbon monoxide. Lower ambient temperatures exacerbate carbon monoxide release from these heating devices. According to the World Bank, about 40% of the urban population uses solid fuels for residential heating [39]. The smell of smoke and burning coal envelops the entire city of Bishkek every winter evening. According to the government, the annual total emissions from stationary sources into the atmosphere of Bishkek is 240,000 tons [38].

Over the past 30 years, the population of Bishkek has doubled, exceeding one million in 2018. The sharp increase in population coincided with the internal migration of rural residents, for which the city was unprepared. With the influx of people to the capital, Bishkek began to build up chaotically outside the initially‐outlined borders. Forty‐seven residential areas have sprung up around the city in recent years. To sustain residents' coal demands, city authorities have encouraged the use of natural gas as an alternative while limiting environmental damage. The Russian corporation Gazprom has recently been engaged in providing natural gas to residential areas in Bishkek, and residents have been urged to abandon solid fuels. However, resistance to this plan has been encountered among the (mostly) poor population in these areas, as heating houses using coal is much cheaper than gas. Every fourth Kyrgyzstani lives below the poverty line, and they are concerned less about the environment than about survival [40].

The use of poor‐quality coal for residential heating has compounded the effects of pollution from densely‐populated areas. Most of the city population has switched to using cheaper coal from the local Kara‐Keche open‐pit mine, which has a high ash content and a relatively low heat capacity [41]. Furthermore, the chaotic development of high‐rise buildings during a recent construction boom, especially in the central and southern parts of the city, which are closer to the mountains, has led to a significant reduction in the natural air corridor from the mountains and harmful smog stagnation. In the 19th century's city design, the streets ran parallel to each other, and the air from the mountains could ventilate the city. However, since the entire foothills area is being built up, there is no such ventilation effect today. The climatic features of the Chui Valley, where the city is located, also contribute to the accumulation of harmful pollution in the lower atmospheric layers, especially during the winter, when temperature inversion sets in [42].

Traffic is another primary source of air pollution in Bishkek. Up to 60%–87% of air pollution is caused by mobile sources in the cold months of the year [43 ^,^ 44]. Annually, vehicles emit 400,000 tons of pollutants. Notably, the emission of SO_2_ per capita in Bishkek was 27 kg in 2018 [38]. There are about 500,000 registered cars in Bishkek, one for every second inhabitant. This is 10 times higher than the city was designed. The preponderance of cars has been driven by the inflow of funds from Kyrgyzstanis that have migrated abroad; cars are among the first purchases of Kyrgyz people who earn money abroad [45 ^,^ 46]. More than 90% of vehicles in Bishkek are over 10 years old, have high emissions, and run‐on low‐quality fuel because of a lack of regulation regarding imported petroleum products. At the same time, technical inspection is required by law for commercial vehicles but not for personal vehicles. Poor public transport has also contributed to the growth in the number of cars. There are not enough trolleybuses and buses in the city; the alternative is overcrowded minibuses that run on diesel fuel [47]. A significant reduction in green park areas also contributes to urban air pollution. Nowadays, the average citizen has 3.5 m^2^ of green space, compared to about 30 m^2^ in the 1980s [48].

### State policy towards improving ambient air quality

Kyrgyzstan ratified the Paris Climate Agreement [49], which provides for the reduction of greenhouse gas emissions into the atmosphere. The government considers climate change to be one of the threats to the environmental safety of the country. The state's approaches and measures to solve the air pollution issues are reflected in its laws and policies. The Law of the Kyrgyz Republic on the protection of atmospheric air, dated June 12, 1999, contains general provisions for the presence of air quality standards; maximum permissible emissions of pollutants and harmful effects; fines for emissions, and air consumption for industrial needs; regulation of emissions from stationary and mobile sources; requirements for the construction and commissioning of facilities; and accounting of harmful effects, observation, and control over the state of the air. The law also defines the types of air pollution violations, as well as measures (criminal, administrative, or other) of responsibility.

The National Development Strategy of the Kyrgyz Republic for 2018–2040, approved by the Decree of the President in November 2018, confirms the country's commitment to achieving the Sustainable Development Goals (SDGs) and green growth [50]. The Strategy's fundamental principle is disease prevention and reduction of health costs. The Kyrgyz government strives to transform Kyrgyzstan into a country with a preserved unique ecosystem, a favourable environment for human life, and efficient use of natural resources for climate‐sustainable development. The Strategy‐2040 key tenets are that a human is at the centre of the country's development and that economic growth should be achieved by minimizing the negative environmental impact. The Strategy includes such measures as raising awareness of environmental issues; green development and climate change adaptation; restoration of the natural environment, landscapes, ecosystems, and biological diversity; ensuring ecological safety; sustainable waste management; revising policy on transport sector development to reduce emissions of pollutants and greenhouse gases; and the gradual transition to environmentally friendly modes of transport by encouraging the use of electric vehicles and bicycles, electrification of railway lines, and development of bicycle infrastructure.

Among the medium‐term goals of the Strategy until 2023 are the reduction of premature mortality from non‐communicable diseases—from cardiovascular diseases by 7.7%, cancer by 8.1%, and diabetes by 8.3%. Other objectives include large‐scale implementation of energy efficiency and energy savings programmes, increased power generation capacity based on renewable energy sources by 10% or by 385 MW, construction of one modern gas compressor station and linking households to the natural gas system, reduction of CO_2_ emissions by all stakeholders, creation of a new landfill based on newly‐developed technologies, an increase in green spaces, reconstruction of road transport infrastructure, expansion of cycling lanes, increased tax rates and fees on older vehicles, an information campaign on measures to improve the environmental situation in the city, and construction of a plant for electric vehicle assembly and production.

The Development Program of the Kyrgyz Republic for 2018–2022, “Unity, Trust, and Creation,” was developed to address numerous key challenges [44]. It focuses on safety and security from threats to health, including climate change and environmental degradation caused by human activities. In this regard, the government is working on updating, and modernizing the public healthcare system, as well as providing sufficient funding and competent staff. Within this programme, the government has been improving the existing environmental data management in order to have a solid base upon which to reasonably formulate and implement development plans, as well as to make environmentally‐relevant decisions. In particular, the environmental impact assessment system should be a permanent component of national policy, ensuring strategic environmental assessments of programmes, legislative acts, and economic and investment projects. This system contains measures to respond to existing and potential risks in the form of man‐made disasters and climate change, and identify steps for mitigation and adaptation. Another environmental focus is on the rational use of natural resources by introducing and promoting resource‐saving green technologies.

In the field of transport, the programme aims to achieve clean air and clean transport as an integral part of the urban environment. According to it, the urban space should be developed not for cars but for people and public transport. This may pave the way for greener transport policies in other Kyrgyzstani cities. In 2021, a Plan was developed to improve the environment in Bishkek based on the mentioned Strategy‐2040 [51]. The plan includes the development of legal acts regulating the state control and monitoring air pollution, construction of a new sanitary landfill and a plant for sorting solid household waste, the use of gas and other alternative heating systems, the creation of a laboratory to control the quality of coal, the development of the transport sector to reduce emissions, a gradual transition to environmentally‐friendly modes of transport, electrification of railway lines, the development of bicycle paths, expansion of parks and greening of streets, and other measures.

### Perspectives of research and policy to improve air quality in Kyrgyzstan

The state's approaches to solve the air pollution issue they are taken recently and there is no effect seen from the actions yet. The realisation of the Strategy‐2040 needs in the support from academic community, mass media, residents and other stakeholders. The state public health statistics needs deeper scientific cause‐effect analysis. There insufficient research activity on adverse health effect of air pollution in Bishkek and Kyrgyzstan. Such research findings can serve as a tool for evidence‐based decision making to improve people's health. A series of training of environmental health researchers by world‐class experts is needed to enhance the quality of research. In collaboration with epidemiologists, economists should study the economic cost of air pollution in Bishkek. It will allow for a cost‐benefit analysis and arguments in favour of specific green policy measures.

Policies, communications, financial incentives, and legal regulation should be built so that citizens prefer to walk in a clean green Bishkek, ride bicycles or use environmentally friendly transport and public infrastructure. By own examples of environmentally friendly behaviour and thinking, the authorities should introduce a new lifestyle and behaviour, production, and consumption patterns for all government agencies, citizens, and businesses. It is necessary to motivate through financial incentives and measures of legal responsibility, as well as encourage them to consciously choose environmentally friendly behaviour models. For example, it is possible to stimulate residents to transit from heating with coal to electric heating by introducing differentiated electric tariffs: “winter”—as low as possible, “summer”—increased, so that electric heating would not be so expensive for households in winter compared to coal. The Bishkek community needs in regular information of on the urban air quality and the progress of green projects. Bishkek authorities should carefully study the experience of the cities in the world that experienced environmental issues and achieved great results in restoration of healthy urban ecology. The “trodden path” is a way to reduce financial costs, and to preserve public health.

## CONCLUSION

The state's approaches to solving the problem of air pollution are correct. However, implementing plans to improve the air environment is costly for a country with a small budget. Success is impossible without active assistance, participation, and monitoring by the public and the non‐governmental sector to formulate and implement state policy on clean air issues. When planning and implementing air quality programs in Bishkek, the authorities must introduce a new way of life and models of behaviour, production, and consumption for all public agencies, citizens, and businesses. It requires motivation through financial incentives and measures of legal responsibility and an incentive to consciously choose an environmentally‐friendly model of behaviour. As a result of the measures taken by the authorities of Kyrgyzstan and the city of Bishkek, public health and the environment—particularly the quality of ambient air—should dramatically improve.

## AUTHOR CONTRIBUTIONS

Kenesh O. Dzushupov: directed the project, and wrote the paper with input from all authors; responded to reviewers. Julian M.A. Buban: contributed to the manuscript by conceptualizing and methodological approach; approved the final version of the manuscript. Arsen A. Aidaraliev: devised the project and gave the main conceptual idea; approved the final version of the manuscript. Attaullah Ahmadi: made a substantial contribution to the concept of the article; approved the final version of the manuscript. Priyanka Chahal: contributed by data collection; helped with the response to reviewers. Approved the final version of the manuscript. Muiz Ibrahim: contributed by data collection; approved the final version of the manuscript Xu Lin: contributed by analysis of data; approved the final version of the manuscript. M.B.N. Kouwenhoven: contributed by formal analysis, and final revision of the manuscript, and; helped with the response to reviewers.

## CONFLICT OF INTEREST

K.O.D., J.M.A.B., A.A., X.L., and M.B.N.K. are Editorial Board members of Public Health Challenges and co‐authors of this article. They were excluded from editorial decision‐making related to the acceptance of this article for publication in the journal.

## ETHICAL APPROVAL

Not required.

## Data Availability

Data used for the article is available upon request.

## References

[1] Kessel A . Chapter 1 “Early conceptions of air and health”. 2011.

[2] Health Effects Institute . State of Global Air 2020. Special Report. Health Effects Institute;2020.

[3] Almetwally AA , Bin‐Jumah M , Allam AA . Ambient air pollution and its influence on human health and welfare: an overview. Environ Sci Pollut Res. 2020;27:24815‐24830. Accessed 08.01.2022. 10.1007/s11356-020-09042-2 32363462

[4] Lee K , Greenstone M , AQLI Annual Update, September 2021. Accessed 07.01.2022 https://aqli.epic.uchicago.edu/wp‐content/uploads/2021/08/AQLI_2021‐Report.EnglishGlobal.pdf

[5] Meghji J , Mortimer K , Agusti A , et al. Improving lung health in low‐income and middle‐income countries: from challenges to solutions. Lancet. 2021;397(10277): 928‐940. Accessed 08.01.2022. doi:10.1016/S0140‐6736(21)00458‐X. Epub 2021 February 22. PMID: 33631128. 33631128 10.1016/S0140-6736(21)00458-X

[6] Zhang J , Ren D , Cao X , et al. Ambient air pollutants and hospital visits for pneumonia: a case‐crossover study in Qingdao, China. BMC Public Health. 2021;21(66). Accessed 08.01.2022. doi:10.1186/s12889-020-10065-0 PMC7791776 33413265

[7] Nhung NTT , Amini H , Schindler C , et al. Short‐term association between ambient air pollution and pneumonia in children: a systematic review and meta‐analysis of time‐series and case‐crossover studies. Environ Pollut. 2017;230:1000‐1008. Accessed 08.01.2022 doi:10.1016/j.envpol.2017.07.063. Epub 2017 July 25. PMID: 28763933. 28763933

[8] Ratajczak A , Badyda A , Czechowski PO , Czarnecki A , Dubrawski M , Feleszko W . Air pollution increases the incidence of upper respiratory tract symptoms among polish children. J Clin Med. 2021;10(10): 2150. Accessed 07.01.2022. doi:10.3390/jcm10102150 34065636 PMC8156299

[9] HEI Collaborative Working Group on Air Pollution, Poverty, and Health in Ho Chi Minh City , Le TG , Ngo L , et al., Effects of short‐term exposure to air pollution on hospital admissions of young children for acute lower respiratory infections in Ho Chi Minh City, Vietnam. Res Rep Health Eff Inst. 2012(169): 5‐72; discussion 73‐83. Jun. PMID: 22849236. Accessed 07.01.2022. 22849236

[10] Cortes‐Ramirez J , Wilches‐Vega JD , Paris‐Pineda OM , Rod JE , Ayurzana L , Sly PD , Environmental risk factors associated with respiratory diseases in children with socio‐economic disadvantage. Heliyon. 2021;7(4):e06820. Accessed 07.01.2022 doi:10.1016/j.heliyon.2021.e06820. PMID: 33997379. 33997379 PMC8093469

[11] Reilly JP , Zhao Z , Shashaty MGS , et al. Low to moderate air pollutant exposure and acute respiratory distress syndrome after severe trauma. Am J Respir Crit Care Med. 2019;199(1): 62‐70. Accessed 08.01.2022. doi:10.1164/rccm.201803-0435OC. PMID: 30067389. 30067389 PMC6353017

[12] Bellani G , Laffey JG , Pham T , et al. Epidemiology, patterns of care, and mortality for patients with acute respiratory distress syndrome in intensive care units in 50 countries. JAMA. 2016;315(8): 788‐800. Accessed 08.01.2022. doi:10.1001/jama.2016.0291 26903337

[13] Zhang Z , Zhu D , Cui B , et al. Association between particulate matter air pollution and lung cancer. Thorax. 2020;75:85‐87. Accessed 08.01.2022. doi:10.1136/thoraxjnl-2019-213722 31727788

[14] Sapunar‐Zenteno J , Ferrer‐Rosende P , Caglevic C , Incidence of lung cancer and air pollution in boroughs of Chile: an ecological study. Ecancermedicalscience. 2021;15:1247. Published 2021 June 10. Accessed 08.01.2022 10.3332/ecancer.2021.1247 34267803 PMC8241455

[15] Kaufman JD , Adar SD , Barr RG , et al. Association between air pollution and coronary artery calcification within six metropolitan areas in the USA (the Multi‐Ethnic Study of Atherosclerosis and Air Pollution): a longitudinal cohort study. Lancet. 2016;388(10045): 696‐704. Accessed 08.01.2022. 10.1016/S0140-6736(16)00378-0 27233746 PMC5019949

[16] Brunekreef B , Hoffmann B . Air pollution and heart disease. Lancet, 388, 10045, 640‐642. Accessed 08.01.2022 doi:10.1016/S0140‐6736(16)30375‐0 10.1016/S0140-6736(16)30375-0 27233744

[17] Desikan A , Crichton S , Hoang U , et al. Effect of exhaust‐ and nonexhaust‐related components of particulate matter on long‐term survival after stroke. J Stroke. 2016;47(12): 2916‐2922. Accessed 08.01.2022. doi:10.1161/STROKEAHA.116.014242 27811334

[18] Lee KK , Miller MR , Shah ASV . Air pollution and stroke. J Stroke. 2018;20(1):2‐11. 10.5853/jos.2017.02894 29402072 PMC5836577

[19] Oudin A , Forsberg B , Kristina J . Air pollution and stroke. Epidemiology. 2012;23(3): 505‐506. Accessed 08.01.2022. doi:10.1097/EDE.0b013e31824ea667 22475837 10.1097/EDE.0b013e31824ea667

[20] Hong Y‐Ch , Lee J‐T , Kim H , and Kwon H‐J . Air pollution: a new risk factor in ischemic stroke mortality. J Stroke. 2002;33(9). Accessed 08.01.2022. doi:10.1161/01.STR.0000026865.52610.5B 10.1161/01.str.0000026865.52610.5b 12215581

[21] Mazidi M , Speakman J . Impact of obesity and ozone on the association between particulate air pollution and cardiovascular disease and stroke mortality among US adults. JAHA. 2018;7:11. Accessed 08.01.2022. doi:10.1161/JAHA.117.008006 PMC6015356 29848499

[22] Wang X , Liu C , Zhang M , et al. Evaluation of maternal exposure to PM2.5 and its components on maternal and neonatal thyroid function and birth weight: a cohort study. Thyroid. 2019;29:1147‐1157. Accessed 08.01.2022 10.1089/thy.2018.0780 31298631

[23] Ghassabian A , Pierotti L , Basterrechea M , et al. Association of exposure to ambient air pollution with thyroid function during pregnancy. JAMA Netw Open. 2019;2(10): e1912902. Accessed 09.01.2022. doi:10.1001/jamanetworkopen.2019.12902. PMID: 31617922 31617922 PMC6806433

[24] Ilias I , Kakoulidis I , Togias S , et al. Atmospheric pollution and thyroid function of pregnant women in Athens, Greece: a pilot study. Med Sci (Basel). 2020;8(2): 19. Accessed 07.01.2022. doi:10.3390/medsci8020019. PMID: 32260367. 32260367 PMC7353503

[25] Block ML , Calderón‐Garcidueñas L , Air pollution: mechanisms of neuroinflammation and CNS disease. Trends Neurosci. 2009;32(9):506‐516. Accessed 08.01.2022 doi:10.1016/j.tins.2009.05.009. Epub 2009 August 26. PMID: 19716187. 19716187 PMC2743793

[26] Genc S , Zadeoglulari Z , Fuss SH , Genc K . The adverse effects of air pollution on the nervous system. J Toxicol. 2012;2012: 782462. Accessed 08.01.2022. 10.1155/2012/782462. Epub 2012 February 19. PMID: 22523490. 22523490 PMC3317189

[27] Hahad O , Lelieveld J , Birklein F , Lieb K , Daiber A , Münzel T . Ambient air pollution increases the risk of cerebrovascular and neuropsychiatric disorders through induction of inflammation and oxidative stress. Int J Mol Sci. 2020;21(12): 4306. Accessed 08.01.2022. doi:10.3390/ijms21124306. PMID: 32560306. 32560306 PMC7352229

[28] Grandjean P , Landrigan PJ . Neurobehavioural effects of developmental toxicity. Lancet Neurol. 2014;13:330‐338. Accessed 07.01.2022. doi:10.1016/S1474-4422(13)70278-3 24556010 PMC4418502

[29] World Health Organization . (2021). WHO global air quality guidelines: particulate matter (PM2.5 and PM10), ozone, nitrogen dioxide, sulfur dioxide, and carbon monoxide. Accessed 04.08.2022. https://www.iqair.com/ru/blog/air‐quality/2021‐WHO‐air‐quality‐guidelines 34662007

[30] Public health and healthcare in the Kyrgyz Republic in 2015–2019, National Statistical Committee, 2020.

[31] Ten EE , Sharshenova AA . Hygienic assessment of the impact of atmospheric air pollution on the health status of children in Bishkek. Med Kyrgyzstan. 2008;3:63‐65.

[32] Samigullina AE , Torеgeldieva ChB , Nazaralieva SB . Somatic pathology and complications of gestation in Bishkek women: the predictive significance of risks of atmospheric air pollution. Int J Appl Basic Med Re. 2019;9:39‐45.

[33] The WHO . The global health observatory. Ambient air pollution attributable DALYs. Accessed 09.01.2022 https://www.who.int/data/gho/data/indicators/indicator‐details/GHO/ambient‐air‐pollution‐attributable‐deaths

[34] ChJ Murray , L , et al. Global burden of 87 risk factors in 204 countries and territories, 1990–2019: a systematic analysis for the Global Burden of Disease Study 2019. Lancet. 2020;396:1223‐1249. Accessed 08.01.2022. 10.1016/S0140-6736(20)30752-2 33069327 PMC7566194

[35] UNDP convenes stakeholders to accelerate efforts addressing air pollution in Kyrgyzstan. Press‐release. December 2, 2019. Accessed 22.03.2022 https://www.undp.org/kyrgyzstan/press‐releases/undp‐convenes‐stakeholders‐accelerate‐efforts‐addressing‐air‐pollution‐kyrgyzstan

[36] WHO Regional Office for Europe . Economic cost of the health impact of air pollution in Europe: Clean air, health and wealth. Copenhagen: WHO Regional Office for Europe. OECD;2015. Accessed 22.03.2022. https://www.euro.who.int/__data/assets/pdf_file/0004/276772/Economic‐cost‐health‐impact‐air‐pollution‐en.pdf

[37] The Lancet Commission on pollution and health , 2017, The Lancet 2017: supplementary appendix, Accessed 22.03.2022 https://www.thelancet.com/journals/lancet/article/PIIS0140‐67361732345‐0/fulltext?code=lancet‐site#sec1

[38] Sabyrbekov R . Economic costs of air pollution in Bishkek, OSCE Academy, 2021. The presentation of the Conference “Air quality in Bishkek and ways to solve the problem” as part of the Green Economy Days in the Kyrgyz Republic October 20, 2021. Accessed 02/08/2022 https://avzur.kg/?p=351&lang=en

[39] The World Bank . KEEPING WARM: urban heating options in the Kyrgyz Republic. Summary report, March 2015. Accessed 07.01.2022 http://documents1.worldbank.org/curated/en/555021468011161504/pdf/97409‐WP‐P133058‐Box391503B‐PUBLIC‐Heating‐Assessment‐for‐Kyrgyz‐P133058‐Final.pdf

[40] The World Bank . Global poverty working group. Accessed 07.01.2022 https://data.worldbank.org/indicator/SI.POV.NAHC?locations=KG

[41] Karabev CO , Lokshina IM , Gainullina IP , et al. Technical characteristics of Kara‐Keche coal. Vestnik KRSU. 2010;10(10): 158‐160. Accessed 22.03.2022. https://www.elibrary.ru/item.asp?id=21788816

[42] Podrezov О , Podrezov А , Riazanov VE . Bishkek city atmospheric air pollution in the winter season of 2017–2018. Vestnik KRSU. 2018;18(12): 126‐133. Accessed 07.01.2022. http://vestnik.krsu.edu.kg/archive/24/754

[43] State Agency for Environmental Protection and Forestry . Environment in the Kyrgyz Republic in 2011–2015. 2016. Accessed 22.03.2022. http://stat.kg/media/publicationarchive/8c0e9d22‐6bb6‐4145‐b1d6‐8311da33521d.pdf

[44] Health and pollution action plan . The Kyrgyz Republic. 2019. UNIDO. Accessed 22.03.2022 https://www.unido.org/sites/default/files/files/2019‐10/Kyrgyzstan%20HPAP.English.pdf

[45] Muktarbek kyzy A , Seyitov Ch and Jenish N . Impact of remittances on the structure of household expenditures in the Kyrgyz Republic. National Bank of the Kyrgyz Republic, Center for Economic Research. 2015:2. Accessed 09.01.2022. https://www.nbkr.kg/DOC/20012016/000000000039978.pdf

[46] Zhanybek kyzy L , Toktakunov A , Where do migrants' remittances go? Radio Freedom. 22.06.2015. Accessed 09.01.2022 https://rus.azattyk.org/a/27084968. https://kloop.kg/blog/2018/08/23/strana‐na‐izhdivenii‐kyrgyzstan‐vyzhivaet‐na‐dengi‐migrantov‐no‐ne‐umeet‐ih‐tratit/

[47] JICA urban transport improvement study in Bishkek, Kyrgyz Republic. Final report, 180 p. Accessed 09.01.2022 https://openjicareport.jica.go.jp/pdf/12127627_02.pdf

[48] Busurmankulova AO . Present condition of greening in Bishkek city. Izvestia vuzov Kyrgyzstana. 2018;8:66‐69. Accessed 07.01.2022. http://www.science‐journal.kg/media/Papers/ivk/2018/8/66‐69.pdf

[49] PF “Kloop‐Media” News website . Accessed 07.01.2022 https://kloop.kg/blog/2019/11/13/kyrgyzstan‐ratifitsiroval‐parizhskoe‐soglashenie‐o‐klimate‐obyasnyaem‐chto‐eto‐znachit/

[50] The National Development Strategy of the Kyrgyz Republic . The Ministry of Foreign Affairs of the Kyrgyz Republic. Accessed 07.01.2022 http://mfa.gov.kg/en/osnovnoe‐menyu/vneshnyaya‐politika/gosudarstvennye‐programmy/nacionalnaya‐strategiya‐razvitiya‐kyrgyzskoy‐respubliki‐na‐2018‐2040‐gody

[51] The Government of the Kyrgyz Republic . Accessed 07.01.2022 https://www.gov.kg/ru/post/s/utverzhden‐plan‐kompleksnykh‐mer‐po‐uluchsheniyu‐ekologicheskoy‐situatsii‐v‐gorode‐bishkek‐i‐sokulukskom‐alamudunskom‐rayonakh‐chuyskoy‐oblasti‐na‐2021‐2023‐gody

